# Fine-tuning of microglia polarization prevents diabetes-associated cerebral atherosclerosis

**DOI:** 10.3389/fimmu.2022.948457

**Published:** 2022-07-22

**Authors:** Xuan Zhu, Pengfei Xing, Ping Zhang, Minmin Zhang, Hongjian Shen, Lei Chen, Fang Shen, Yi Jiang, Hui Yuan, Lei Zhang, Jing Wang, Xiongfeng Wu, Yu Zhou, Tao Wu, Benqiang Deng, Jianmin Liu, Yongwei Zhang, Pengfei Yang

**Affiliations:** Department of Neurovascular Center, Changhai Hospital, Naval Medical University, Shanghai, China

**Keywords:** microglia, polarization, cerebral atherosclerosis, interleukin-10 (IL-10), TMEM119

## Abstract

Diabetes increases the occurrence and severity of atherosclerosis. When plaques form in brain vessels, cerebral atherosclerosis causes thickness, rigidity, and unstableness of cerebral artery walls, leading to severe complications like stroke and contributing to cognitive impairment. So far, the molecular mechanism underlying cerebral atherosclerosis is not determined. Moreover, effective intervention strategies are lacking. In this study, we showed that polarization of microglia, the resident macrophage in the central nervous system, appeared to play a critical role in the pathological progression of cerebral atherosclerosis. Microglia likely underwent an M2c-like polarization in an environment long exposed to high glucose. Experimental suppression of microglia M2c polarization was achieved through transduction of microglia with an adeno-associated virus (serotype AAV-PHP.B) carrying siRNA for interleukin-10 (IL-10) under the control of a microglia-specific TMEM119 promoter, which significantly attenuated diabetes-associated cerebral atherosclerosis in a mouse model. Thus, our study suggests a novel translational strategy to prevent diabetes-associated cerebral atherosclerosis through *in vivo* control of microglia polarization.

## Introduction

Atherosclerosis is characterized by lipid accumulation in the arterial vessel wall (1). The formation of atherosclerotic plaques narrows the arterial lumen to increase the risk of myocardial infarction and stroke (2). When the plaques are formed in cerebral vessels, the cerebral atherosclerosis also contributes to development of cognitive impairment (3).

Epidemiological data on cerebral atherosclerosis are currently lacking because of the lack of a standardized and uniform assessing method. Atherosclerosis can lead to various types of stroke, including ischemic and hemorrhagic (4). Atherosclerotic thrombosis is one of the most important causes of ischemic stroke (5). Atherosclerotic populations are at high risk for ischemic stroke. Atherosclerosis mostly occurs in middle-aged people over the age of 40 (6). As the age of the patients increases, the morbidity and mortality also increase accordingly. The male-to-female ratio in all patients is 2:1 (6). However, women are more affected after menopause, when both estrogen and blood high-density lipoprotein (HDL) decrease (6). Europeans and Americans often develop severe cerebral atherosclerosis in the initial segment of the internal carotid artery, while Africans or Asians more commonly develop it in the intracranial artery (6). So far, the molecular mechanism underlying cerebral atherosclerosis has not been determined (7).

Diabetes increases the occurrence and severity of cerebral atherosclerosis (8). Reports have shown that both cervical and cerebral atherosclerosis is more severe in diabetes than that in non-diabetics. In a recent meta-analysis, the 4,019 patients with type 2 diabetes and 1,110 patients with glucose intolerance exhibited greater carotid intima-media thickness than the control patients (9).

Interestingly, as a resident macrophage in the central nerve system, microglia play critical roles in the inflammatory events and act as neuroinflammatory intermediaries between lipid overload and neurodegeneration (10). Moreover, a very recent study showed that diabetic status alters the phenotype of reticular microglia, which contributes to the development of diabetic retinopathy (11). Thus, it may be interesting to assess whether the diabetes-induced polarization of cerebral microglia may affect the pathological progress of cerebral atherosclerosis. This question was thus addressed in the current study.

## Materials and methods

### Protocol approval

All the experimental protocols including animal work have been approved by the research committee and Institutional Animal Care and Use Committee at Changhai Hospital. A power calculation (p < 0.05) was done to determine the number of animals. Randomization was used for allocation.

### Animal, diabetes, and AAV transplantation

ApoE (−/−) mice were purchased from SLAC Laboratory Animal (Shanghai, China) and maintained under standard animal room conditions (20 ± 2°C and 56% humidity). Male and female mice were evenly distributed in each experimental group. Diabetes was induced in 10-week-old ApoE (−/−) mice by single intraperitoneal (i.p.) injection of 120 mg/kg streptozotocin (STZ) in 100 µl normal saline after an overnight fasting. The control mice received 100 µl normal saline. Fasting blood glucose was determined after an overnight fasting. Beta-cell mass was assessed based on percentage of the insulin+ area to the total pancreatic area multiplied by the pancreatic weight. AAVs (10^11^ in 100 µl) were intravenously (i.v.) injected through the tail vein. Four groups of ApoE (−/−) mice were included in this study: group 1: mice received i.p. saline only; group 2: mice received i.p. STZ; group 3: mice received i.p. STZ and i.v. control AAVs; group 4: mice received i.p. STZ and i.v. experimental AAVs. The analysis was done after 24 weeks.

### Measurement of cerebral blood flow

Quantitative cerebral blood flow (CBF; ml/g/min) was assessed with an MRI-based continuous arterial spin labeling technique. Briefly, a horizontal magnet and a BGA12S gradient insert were applied in the measurement. At mouse heart, a circular surface coil and a circular labeling coil were used for continuous arterial spin labeling. CBF was obtained by the affiliated software with the apparatus.

### Cell culture and AAV production

Microglia cell line HMC3, macrophage cell line KG-1, neuronal cell line HCN-2, and fibroblast cell line 3T3 were all purchased from the American Type Culture Collection (Rockville, MD, USA) and cultured in RPMI 1640 media supplemented with 7.5% fetal bovine serum (FBS, Invitrogen, CA, Carlsbad, USA) and 1% penicillin/streptomycin (Invitrogen) at 37°C with 5% CO_2_. Transfection of human embryonic kidney 293-T cells was done using AAV serotype AAV-PHP.B vectors. The TMEM119 promoter was generated according to published sequences. The sequence for the target sites for IL-10 is 5′-GTCTTCTGGAGTTCCGTTT-3’. The sequence for the control scramble sequence is 5’-GCGCCATTTAAAGTAGGCC-3’. The transfection was performed with Lipofectamine 3000 Reagent (Invitrogen).

### ELISA

The total protein was extracted from cultured cells and underwent analysis using corresponding mouse enzyme-linked immunosorbent assay (ELISA; R&D Systems, Los Angeles, CA, USA). The microplates were read at 450 nm within 40 min.

### Immunocytochemistry and histology

At sacrifice, the dissected pancreas and brain of the mice were fixed in 4% paraformaldehyde (PFA, Invitrogen) for 4 h, followed by an incubation in 30% sucrose for 36 h. The samples were then frozen, embedded, and sectioned at 6 µm. Virus-transduced cells were detected by direct fluorescence of GFP. Immunofluorescent staining for insulin and IL-10 was performed with a guinea pig polyclonal antibody against insulin (Ab7842, Abcam, St. Louis, MO, USA) and a rabbit polyclonal antibody against IL-10 (Ab34843, Abcam), respectively. DAPI (4′,6-diamidino-2-phenylindole) (Sigma-Aldrich, Shanghai, China) was used to stain the nucleus. Atherosclerotic lesions of the aortic root were examined by H&E staining. Quantification of the images was done using NIH ImageJ software (Bethesda, MD, USA).

### Flow cytometry

Mouse brain tissue was digested with 0.25% Trypsin (Invitrogen) and 5 mg/ml DNase (Invitrogen) for 35 min, after which the dissociates passed a 70-µm filter and were briefly fixed and penetrated for preparation for flow cytometry-based cell sorting. GFP was used to detect transduced microglia, and neuronal cells were labeled with an Alexa Fluor^®^ 594-conjugated Rabbit monoclonal antibody against NeuN (ab207279, Abcam).

### Bioinformatics

A Gene Expression Omnibus (GEO, http://www.ncbi.nlm.nih.gov/geo/) database (GSE139276) was analyzed with R package. Metascape (http://metascape.org) was used for pathway enrichment analyses.

### Statistical analysis

Student’s T-test was used to compare two groups. The figures were generated using individual values when possible (GraphPad Software, version 7, Inc., La Jolla, CA, USA). A value of p < 0.05 was considered statistically significant and shown as a *. No significant comparisons were presented as ns.

## Results

### Diabetes alters gene expression in microglia

**Figure 1:**
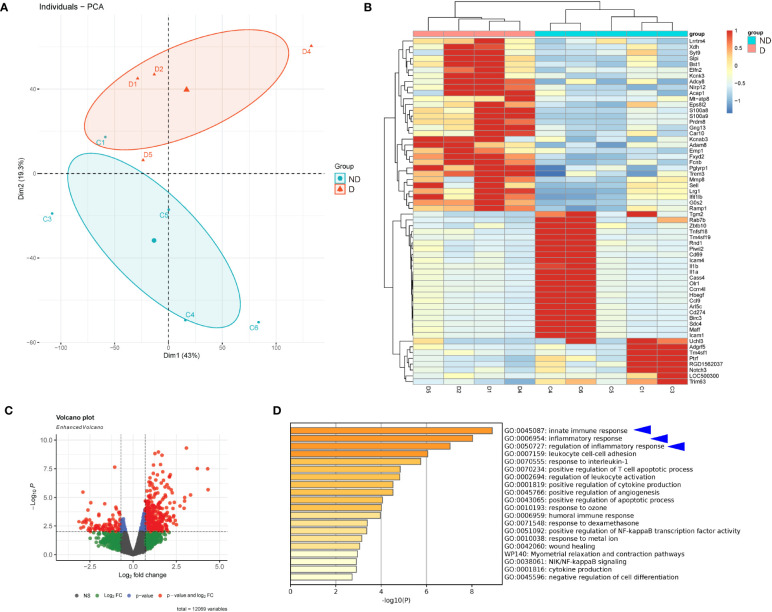
Diabetes alters gene expression in microglia. Bioinformatic analysis on a study, in which the reticular microglia isolated from diabetic (4 weeks) versus non-diabetic mice were compared (GEO139276). **(A)** The principal component analysis (PCA) plot. **(B, C)** Heat map **(B)** and volcano plot map **(C)** to show the differentiated genes. **(D)** Pathway analysis showing that the most different expressing gene clusters between the reticular microglia from diabetic versus non-diabetic mice were innate immune response, inflammatory response, and regulation of inflammatory response (blue arrowheads).

In order to assess the alteration in gene expression in microglia under a diabetic status, we first explored the published database at GEO. From the very limited available sources, we found one interesting study, in which the reticular microglia were isolated from diabetic (4 weeks) versus non-diabetic mice (GEO139276). Thus, we performed a bioinformatics analysis on this database. The principal component analysis (PCA) demonstrated similarities among samples in the same group (**Figure 1A**). Differential genes were shown in a heat map (**Figure 1B**) or in a volcano plot map (**Figure 1C**). Interestingly, by pathway analysis, we found that the most different expressing gene clusters between the reticular microglia from diabetic versus non-diabetic mice were innate immune response, inflammatory response, and regulation of inflammatory response, which were all involved in microglia polarization (**Figure 1D**).

### Diabetes alters microglia polarization in a complicated way

**Figure 2:**
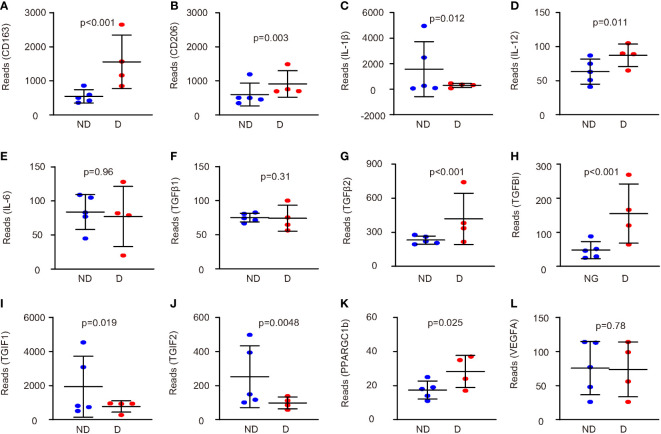
Diabetes alters microglia polarization in a complicated way. **(A–L)** The genes related to microglia polarization were examined in this database (GEO139276). Comparisons of expression levels of CD163 **(A)**, CD206 **(B)**, IL-1β **(C)**, IL-12 **(D)**, IL-6 **(E)**, TGFβ1 **(F)**, TGFβ2 **(G)**, TGFBI **(H)**, TGIF1 **(I)**, TGIF2 **(J)**, PPARGC1b **(K)**, and VEGF-A **(L)**. p values were shown.

Next, we examined the genes that were related to microglia polarization in this database. Macrophages and microglia are typically catalogized into pro-inflammatory M1 and anti-inflammatory M2 phenotype (12). CD163 and CD206 were two markers for anti-inflammatory M2 microglia markers, and they were both increased in microglia from mice that had developed diabetes for 4 weeks (**Figures 2A, B**). We did not find data for reactive oxygen species (ROS), nitric oxide synthase (iNOS), two key M1 markers, or arginase 1 (ARG1), a M2 marker, in this database. However, when we analyzed three M1 cytokines, IL-1β, IL-12, and IL-6, we found that diabetes microglia had significantly lower levels of IL-1β (**Figure 2C**), significantly higher levels of IL-12 (**Figure 2D**) and unaltered levels of IL-6 (**Figure 2E**). The paradox data suggest that the diabetes may alter microglia polarization in a complicated way, which is not a simple M1/M2 direction. Since M2 microglia could be further sub-catalogized into M2a (traditional M2), M2b (close to M1 with some pro-inflammatory effects), M2c (express high levels of IL-10 and TGFβ to be associated with tissue remodel and fibrosis), and M2d (express high VEGF-A to be associated with angiogenesis) (13), we thus examined the genes related to this subgroup determination. We did not detect significant changes in TGFβ1 between two groups (**Figure 2F**). However, we detected significant increases in TGFβ2 in diabetic microglia (**Figure 2G**). Moreover, transforming growth factor-induced protein (TGFBI) also significantly increased in diabetic microglia (**Figure 2H**), while two TGFβ inhibitors, TG-interacting factor 1 (TGIF1) and TGIF2, significantly decreased in diabetic microglia (**Figures 2I, J**). Furthermore, another factor (peroxisome proliferator-activated receptor gamma coactivator 1-beta, PPARGC1b) associated with microglia polarization was significantly changed (**Figure 2K**), but VEGF-A was not altered (**Figure 2L**). Together, these data suggest that diabetes (4 weeks) may alter microglia polarization toward an M2c and M2b phenotype.

### Hyperglycemia progressively induces microglia polarization likely to M2c

Based on the analysis on a public database, we thus examined the polarization-related genes in microglia cultured in normal glucose (2.8 mmol/l) or high glucose (16.7 mmol/l) *in vitro*. HMC3 microglia were used and analyzed at start of the culture, 4 weeks’ culture, and 16 weeks’ culture.

**Figure 3:**
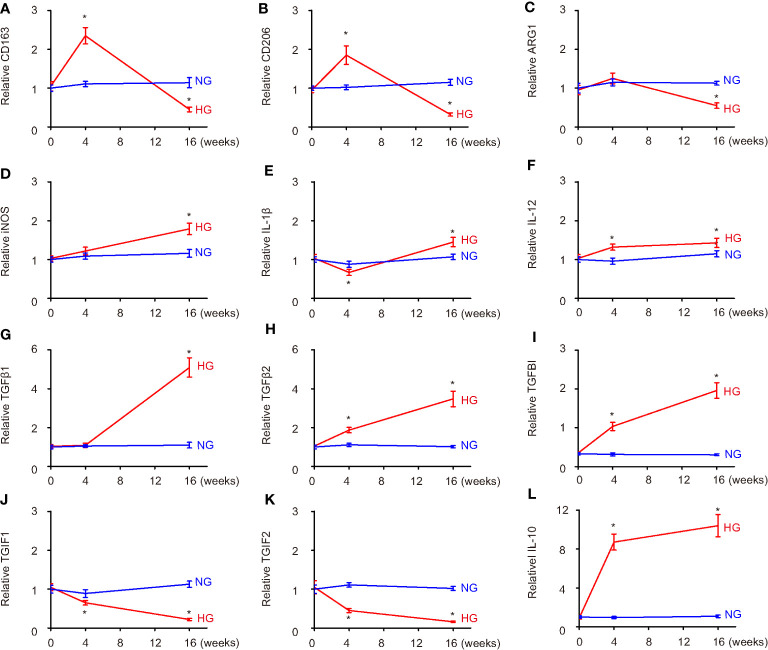
Hyperglycemia progressively induces microglia polarization likely to M2c. **(A–L)** The polarization-related genes were examined in HMC3 microglia cultured in normal glucose (2.8 mmol/l) or high glucose (16.7 mmol/l) *in vitro* at the start of the culture, 4 weeks’ culture, and 16 weeks’ culture. ELISA comparisons of expression levels of CD163 **(A)**, CD206 **(B)**, ARG1 **(C)**, iNOS **(D)**, IL-1β **(E)**, IL-12 **(F)**, TGFβ1 **(G)**, TGFβ2 **(H)**, TGFBI **(I)**, TGIF1 **(J)**, TGIF2 **(K)**, and IL-10 **(L)**. *p < 0.05. N = 5.

We found that high-glucose culture first induced CD163 and CD206 at 4 weeks, but the levels of CD163 and CD206 significantly decreased at 16 weeks (**Figures 3A, B**). ARG1 levels did not significantly change at 4 weeks but significantly decreased at 16 weeks (**Figure 3C**). These data suggest that sustained high-glucose exposure may change microglia into a more proinflammatory phenotype. This conclusion was further supported by analysis on M1 markers NOS, iNOS, IL-1β, and IL-12. Although the changes in the levels of these markers differed at 4 weeks, they all significantly increased at 16 weeks (**Figures 3D–F**, **Supplementary Figure 1**), suggesting polarization of microglia toward a more proinflammatory phenotype in a long-term high-glucose environment. The analysis on TGFβ1, TGFβ2, TGFBI, TGIF1, and TGIF2 suggested a continuous trend to favor M2c polarization (**Figures 3G–K**), which was further supported by analysis on an M2c trigger, IL-10 (**Figure 3L**). Together, these data suggest that long exposure to high glucose may alter microglia polarization toward a consistent M2c and an early M2b/late M1 phenotype.

### Generation of AAVs that specifically deplete IL-10 in microglia

**Figure 4:**
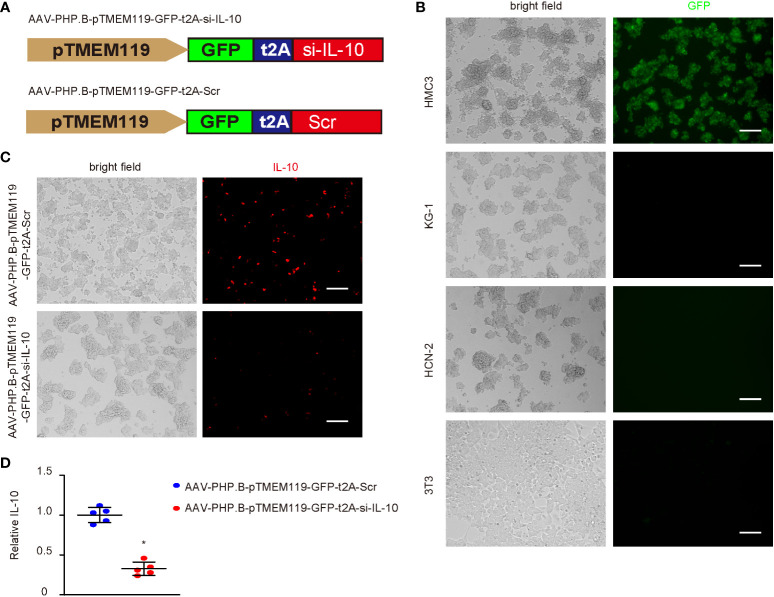
Generation of AAVs that specifically deplete IL-10 in microglia. **(A)** Generation of an AAV PHP.B serotype carrying siRNA for IL-10 (si-IL-10) or control scrambled sequence (Scr) under a microglia-specific TMEM119 promoter, which allows specific depletion of IL-10 in microglia. T2A allowed co-expression of transgenes with a GFP reporter under the same promoter. **(B)** Scr virus was used to infect four different cell lines, which were microglia cell line HMC3, macrophage cell line KG-1, neuronal cell line HCN-1, and fibroblast cell line 3T3. Bright fields and GFP channel were shown. **(C)** Si-IL-10 and control Scr viruses were used to infect HMC3 cells. Bright fields and immunocytochemistry for IL-10 were shown. **(D)** ELISA for IL-10. *p < 0.05. N = 5. Scale bars are 100 µm.

Since IL-10 is a known M2c trigger, we aimed to see if IL-10 depletion in microglia could prevent or reverse the M2c polarization of diabetes microglia and generate a positive effect on cerebral atherosclerosis. Thus, we generated an AAV carrying siRNA for IL-10 under a microglia-specific TMEM119 promoter, which allows specific depletion of IL-10 in microglia. To allow systemic administration of the AAVs and their penetration into the central nervous system, a specific PHP.B serotype was used. This serotype could pass the blood–brain barrier. A scrambled sequence for si-IL-10 was used as a control (Scr; **Figure 4A**). To ensure the specificity of the TMEM119 promoter, the Scr virus was used to infect four different cell lines, which were microglia cell line HMC3, macrophage cell line KG-1, neuronal cell line HCN-1, and fibroblast cell line 3T3. Only infected HMC3 cells expressed GFP, confirming the microglia-specific activation of the TMEM119 promoter (**Figure 4B**). Si-IL-10 and control Scr viruses were thus used to infect HMC3 cells (**Figure 4C**) and showed significant knockdown of IL-10 by si-IL-10 (**Figure 4D**).

### Depletion of IL-10 in microglia does not alter diabetic status in mice

**Figure 5:**
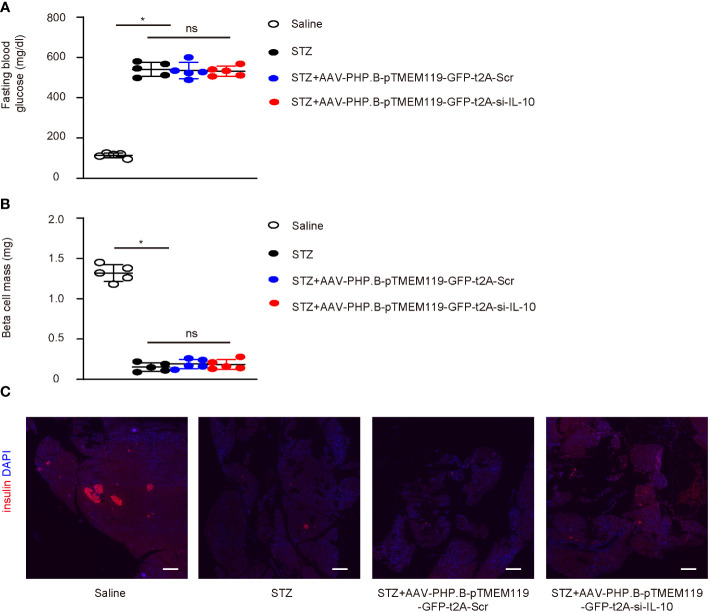
Depletion of IL-10 in microglia does not alter diabetic status in mice. **(A–C)** The effects of depletion of IL-10 in diabetic microglia were then assessed in a mouse model for cerebral atherosclerosis, ApoE (−/−) mice that receive STZ to develop diabetes. Four groups of ApoE (−/−) mice were included in this study: group 1—mice received i.p. saline only; group 2—mice received i.p. STZ; group 3—mice received i.p. STZ and i.v. control AAVs; group 4—mice received i.p. STZ and i.v. experimental AAVs. The analysis was done after 24 weeks. **(A, B)** Fasting blood glucose **(A)** and beta-cell mass **(B)** at analysis (24 weeks). **(C)** Representative immunohistochemistry for insulin and DAPI in pancreas. *p < 0.05. ns: non-significant. N = 5. Scale bars are 200 µm.

The effects of depletion of IL-10 in diabetic microglia were then assessed in a mouse model for cerebral atherosclerosis, ApoE (−/−) mice that receive STZ to develop diabetes. Four groups of ApoE (−/−) mice were included in this study: group 1—mice received i.p. saline only; group 2—mice received i.p. STZ; group 3—mice received i.p. STZ and i.v. control AAVs; group 4—mice received i.p. STZ and i.v. experimental AAVs. The analysis was done after 24 weeks. STZ induced sustained hyperglycemia in ApoE (−/−) mice, while injection of any AAVs did not alter fasting blood glucose (**Figure 5A**) or beta-cell mass (**Figures 5B, C**) at analysis.

### Depletion of IL-10 in microglia significantly attenuates diabetes-associated cerebral atherosclerosis in mice

**Figure 6:**
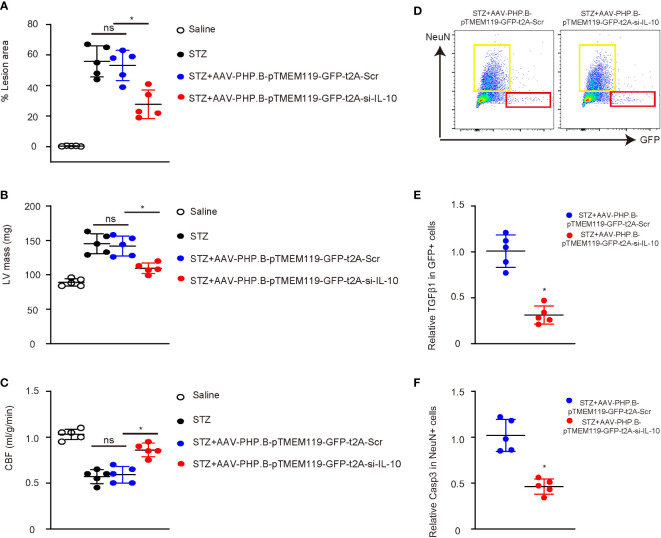
Depletion of IL-10 in microglia significantly attenuates diabetes-associated cerebral atherosclerosis in mice. **(A)** Atherosclerotic lesion size in the aortic arc. **(B)** The weight of left ventricle (LV mass). **(C)** Cerebral blood flow. **(D)** Flow cytometry analysis and sorting of GFP+ cells or NeuN+ cells from mouse brains at analysis. **(E)** ELISA for TGFβ1 in GFP+ microglia. **(F)** ELISA for Caspase 3 (Casp3) in NeuN+ neuronal cells. *p < 0.05. ns: non-significant. N = 5.

Interestingly, we found that depletion of IL-10 in microglia significantly reduced the atherosclerotic lesion size in the aortic arc (**Figure 6A**) and the weight of the left ventricle (**Figure 6B**) and significantly increased the cerebral blood flow (**Figure 6C**). Flow cytometry analysis and sorting of GFP+ cells or NeuN+ cells were thus performed on the mouse brains at analysis (**Figure 6D**). Significantly less TGFβ1 was detected in GFP+ microglia from mice with IL-10 depletion, suggesting that IL-10 depletion suppressed the M2c differentiation of microglia in diabetes (**Figure 6E**). Significantly less Caspase 3 (Casp3) was detected in NeuN+ neuronal cells from mice with IL-10 depletion, suggesting that IL-10 depletion reduced apoptosis of the neuronal cells in diabetes (**Figure 6F**). Hence, depletion of IL-10 in microglia significantly attenuated diabetes-associated cerebral atherosclerosis in mice.

## Discussion

Cerebral atherosclerosis is a chronic disease in the brain arterial system characterized by progressive lipid deposition, augmentation in fibrosis, and inflammatory cell infiltration (14). Large and medium-sized elastic and muscular arteries throughout the body could all be involved. Abnormal lipid metabolism is the most recognized risk factor for atherosclerosis (14). The increase of plasma total cholesterol and triglyceride, of which cholesterol plays a key role, and the oxidative modification of low-density lipoprotein (LDL) are key initiating factors for the formation of atherosclerosis (14). Interestingly, some recent single-cell RNA-sequencing studies have defined specified microglial phenotypes with alterations in the expression of genes involved in lipid and lipoprotein metabolism and related diseases (15–17). Moreover, microglia were recently shown to play critical roles in the inflammatory events and act as neuroinflammatory intermediaries between lipid overload and neurodegeneration (10).

Hypertension is another cause of, and is prone to, atherosclerosis and initiates and accelerates atherosclerosis by damaging the vascular endothelium (18). Microglia mediate neuroinflammation and modulate neuronal excitation, both contributing to the progression of hypertension (19). Thus, microglia play roles in the development of cerebral atherosclerosis in many aspects (20).

Diabetes is a metabolic disorder characterized by hyperglycemia and increases the occurrence and severity of cerebral atherosclerosis (8, 9). High insulin levels stimulate endothelial and smooth muscle cell growth, while high blood sugar and insulin resistance can damage endothelial cells (21). The serum of diabetic patients is rich in vascular endothelial cell adhesion molecules, which are involved in and promote the formation of atherosclerosis (21). However, the role of microglia in cerebral atherosclerosis is much less understood. A very recent study showed that diabetic status alters the phenotype of reticular microglia, which contributes to the development of diabetic retinopathy (11). In this study, reticular microglia were isolated from normoglycemic mice and mice that had developed diabetes for 4 weeks. We analyzed data from this study from the public database and found that microglia with a 4-week diabetic status likely developed a complicated phenotype different from the simple M1/M2. Although diabetic microglia significantly increased their expression of CD163 and CD206, two surface markers for anti-inflammatory M2, they also increased the expression of an M1 marker, IL-12. The other two analyzed M1 markers, IL-1β and IL-6, were either just slightly decreased or remained unchanged. These data did not support a typical M2 polarization. Since M2b is closer to M1, with expression of some pro-inflammatory genes, we think that this diabetes-induced polarization may favor an M2b rather than an M1-to-M2 or M2-to-M1. When we analyzed TGFβ-associated genes, we were astonished that several triggers like TGFβ2 and TGFBI were significantly activated by diabetes, while several suppressors like TGIF1 and TGIF2 were significantly deactivated by diabetes. Since TGFβ is one of the most important triggers and markers of M2c, the remodeling and fibrotic microglia phenotype, we think that the diabetes-induced polarization of microglia may be toward M2c more than that of M2b (22).

Since we did not find readouts of some important genes associated with microglia polarization, we performed an *in vitro* long culture of microglia. The chosen 4-week time point was compared with this published database. We also analyzed a much long time point, 16 weeks, since diabetes or hyperglycemia as a long stimulant may exert further effects on microglia polarization in the long run. We found that although high-glucose culture induced CD163 and CD206 at 4 weeks, the levels of CD163 and CD206 significantly decreased at 16 weeks. A similar finding was obtained from ARG1 levels. On the other hand, M1 markers iNOS, IL-1β and IL-12, all increased at 16 weeks. The loss of M2 markers and obtainment of M1 markers of microglia exposed to high glucose in the long-time culture suggest that although microglia may first adapt to an M2b-like phenotype, they likely turn into M1 in the long run (22). On the other hand, the persistent or even increased expression of TGFβ triggers and the continuous loss of TGFβ suppressors in the long-time culture suggest that the adaption to M2c is likely kept by microglia long exposed to high glucose (22). It is thus clear that high-glucose exposure first polarizes microglia to M2b and M2c and later to M1 and M2c phenotypes.

Due to the tissue remodeling effects of M2c (23–25), we hypothesized it a possible effect of microglia on cerebral atherosclerosis in diabetes. IL-10 is a very potent trigger of M2c and thus inhibited in the interfering experiment, resulting in suppression of features of cerebral atherosclerosis. These functional analyses support our hypothesis that M2c polarization of microglia may be important for diabetes-associated cerebral atherosclerosis. The effects of M2c polarization of microglia on lipid metabolism, hypertension, and neuronal health were not examined in the current study and could be an interesting topic to be explored in the future research.

## Data availability statement

The original contributions presented in the study are included in the article/**Supplementary Material**. Further inquiries can be directed to the corresponding authors.

## Ethics statement

The animal study was reviewed and approved by Changhai Hospital.

## Author contributions

XZ, PX, YZ, and PY are responsible for study conception and design. All authors are responsible for data acquisition and analysis. XZ and PX performed bioinformatics analysis. XZ, PX, YZ, and PY wrote the manuscript, and all authors have read the manuscript and agreed with the publication. YZ and PY are responsible for funding and are the guarantee of the study. All authors contributed to the article and approved the submitted version.

## Funding

This work was supported by National Natural Science Foundation of China (NO:82071278).

## Conflict of interest

The authors declare that the research was conducted in the absence of any commercial or financial relationships that could be construed as a potential conflict of interest.

## Publisher’s note

All claims expressed in this article are solely those of the authors and do not necessarily represent those of their affiliated organizations, or those of the publisher, the editors and the reviewers. Any product that may be evaluated in this article, or claim that may be made by its manufacturer, is not guaranteed or endorsed by the publisher.
