# Supplementary material for: Fine-tuning of microglia polarization prevents diabetes-associated cerebral atherosclerosis
Source: Front Immunol. 2022 Jul 22;13:948457. doi: 10.3389/fimmu.2022.948457 (PMC9353938; doi:10.3389/fimmu.2022.948457)
Supplement: Supplementary file 1 [file Image_1.pdf]

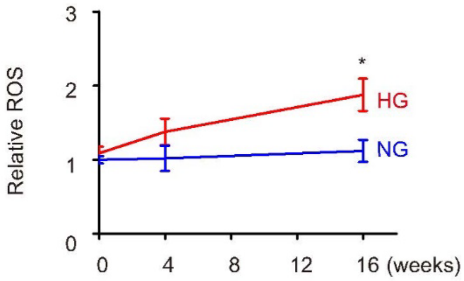

Supplementary Figure 1: Hyperglycemia progressively induces microglia polarization likely to M2c

(A-L) The polarization-related genes were examined in HMC3 microglia cultured in normal glucose (2.8 mmol/l) or high glucose (16.7mmol/l) in vitro at the start of the culture, 4 weeks' culture and 16 weeks' culture. ELISA comparison of expression levels of ROS. *$p<0.05$. N=5.
